# Case Report: Dramatic Response to Crizotinib in a Patient With Non-Small Cell Lung Cancer Positive for a Novel *ARL1-MET* Fusion

**DOI:** 10.3389/fonc.2022.804330

**Published:** 2022-02-14

**Authors:** Qing Ma, Lingping Kong, Diansheng Zhong

**Affiliations:** Department of Oncology, Tianjin Medical University General Hospital, Tianjin, China

**Keywords:** non-small cell lung cancer, *ARL1-MET* fusion, crizotinib, case report, targeted therapy

## Abstract

It is imperative to know the status of oncogenic drivers in patients with non-small cell lung cancer (NSCLC). Compared with *ALK* and *ROS1* fusion, *MET* fusion is relatively rare in NSCLC. In this case, we report the case of a female patient with NSCLC positive for a novel *ARL1-MET* fusion. The patient achieved about a 5-month progression-free survival (PFS) after receiving crizotinib for unresectable right lung malignancies. To the best of our knowledge, this case provides the first clinical evidence that the novel *ARL1-MET* fusion might be an actionable mutation in NSCLC.

## Introduction

Non-small cell lung cancer (NSCLC) is a disease commonly caused by alterations in oncogenic drivers. *MET* fusion is relatively rare compared with *ALK* and *ROS1* fusion in NSCLC. To date, eight *MET* fusion partners have been reported for NSCLC: *ATXN7L1*, *KIF5B*, *STARD3NL*, *SPECC1L*, *HLA-DRB1*, *UBE2H*, *SLC1A2*, and *PTPRZ1*. *MET* fusion has been reported to be a therapeutic target in lung cancer (1–6).

Crizotinib is an orally bioavailable, adenosine triphosphate (ATP)-competitive, small-molecule inhibitor of the receptor tyrosine kinases (RTKs) c-MET [also known as hepatocyte growth factor receptor (HGF)], ALK, and ROS1. When the drug was initially developed, it was mainly used as a small-molecule *MET* inhibitor and has been proven effective in preclinical and phase I clinical trials (7). It can inhibit the proliferation and invasion of HGF-activated endothelial cells, and the antitumor effect is dose dependent, which is closely related to the inhibition of MET phosphorylation (8).

Herein, we reported the case of a 60-year-old woman with NSCLC. This patient was found to have synchronous multiple primary lung cancer (SMPLC), which meant that the time since tumor discovery was less than 6 months. The patient underwent thoracoscopic wedge resection of the left upper lobe and dissection of the mediastinal lymph node. Histologically, postoperative pathology confirmed that the left lung tumor was adenocarcinoma, while the unresectable right lung tumors were confirmed to be adenosquamous carcinomas through bronchoscopic biopsy. A novel *ADP-*ribosylation facto*r*, *GTPase 1 (ARL1)-MET* fusion, was detected in the right lung tumor tissues using next-generation sequencing (NGS). She obtained a 5-month progression-free survival (PFS) after receiving crizotinib for the unresectable right lung malignancies.

## Case Description

**Figure 1:**
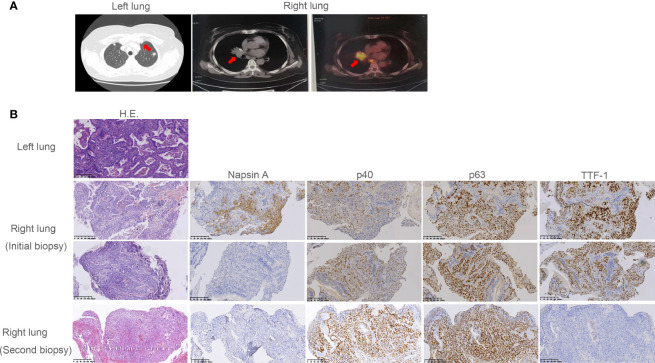
Radiographic imaging at diagnosis and pathological findings. **(A)** The subsolid nodule under the pleura of the upper left lobe and the soft tissue density nodule in the right middle lobe and near the hilum revealed by computed tomography (CT) or positron emission tomography (PET). **(B)** Pathological examination revealed the subsolid nodule under the pleura of the upper left lobe was adenocarcinoma. Pathology showed the initial and the second biopsy of soft tissue density nodule in the right lung were adenosquamous carcinoma and squamous carcinoma, respectively. Interpretation of immunohistochemistry (IHC) performed on the initial biopsy was challenging as most tumor cells were TTF-1 positive suggesting adenocarcinoma. However, there was clear and diffuse positivity for p40 in some cells with corresponding lack of Napsin A. This suggested both glandular and squamous differentiation. In the second biopsy, p40 was diffuse with complete lack of adenocarcinoma markers TTF-1 and Napsin A, consistent with squamous cell carcinoma.

In July 2018, a 60-year-old woman was admitted to our department with complaints of chest tightness and dizziness. She had no other accompanying symptoms such as cough, expectoration, or hemoptysis. She reported a 1-year history of diabetes and was receiving treatment with metformin and voglibose. Thoracic CT showed a subsolid nodule under the pleura of the upper left lobe, approximately 1.1 cm in diameter. The edge of the nodule was rough, indicating a malignant lesion. No metastasis to the head or bone was observed. Of note, thoracic CT showed no nodule in the right lung (**Figure 1A**). In August 2018, the patient underwent thoracoscopic wedge resection of the upper left lobe and mediastinal lymph node dissection. Postoperative pathology revealed that the tumor was an invasive adenocarcinoma without invasion of the pleura (**Figure 1B**). Additionally, lymph node 6 was negative. The patient was diagnosed with stage IA lung adenocarcinoma (pT1bN0M0, **Figure 1B**). Subsequently, she was followed up regularly, and no accompanying symptoms were observed. On December 6, 2018, thoracic CT showed a soft tissue density nodule in the right middle lobe and near the hilum, approximately 20 mm in diameter (**Figure 1A**). On January 25, 2019, thoracic CT revealed that the size of the soft tissue density nodule in the right middle lobe near the hilum had increased to 35 × 29 mm. Meanwhile, multiple enlarged lymph nodules in the right hilum and mediastinum were observed, indicating metastatic lesions. On February 28, 2019, PET/CT showed multiple enlarged lymph nodes in the right neck and chest, as well as a soft tissue mass in the right hilar region. The former were considered to be metastases and the latter a malignant lesion. Subsequently, she underwent a bronchoscopic biopsy. Hematoxylin and eosin (H&E) and immunohistochemical staining of tumor tissues taken from the right lung showed characteristics of adenosquamous carcinoma (**Figure 1B**). Of note, the presence of TTF-1 staining in the p40-positive cells may be due to a less-specific anti-TTF-1 primary antibody (*SPT24*). The tumor in the right lung was confirmed to be stage IVA NSCLC with squamous and glandular differentiation with a favored diagnosis of adenosquamous carcinoma according to the WHO Classification of Tumors, 5th Edition (pT4N3M1a). To seek personalized treatment strategies, formalin-fixed paraffin-embedded (FFPE) sections of tumor tissues from the right lung of the patient were subjected to NGS using a 68-gene panel associated with the pathogenesis and targeted therapy of lung cancer (Burning Rock Biotech, Guangzhou, China) on March 6, 2019. The average sequencing depth is 1,887×. NGS revealed a novel *ARL1-MET* rearrangement (fusion) in the tumor tissue sample from the right lung. The breakpoints occurred within intron 1 of *ARL1* and within exon 14 of *MET*. Therefore, the ARL1-MET fusion protein in the patient was supposed to retain the completed tyrosine kinase domain of *MET*, which conferred potential oncogenic activity (**Figurea 2A, B**; **Table 1**).

**Figure 2:**
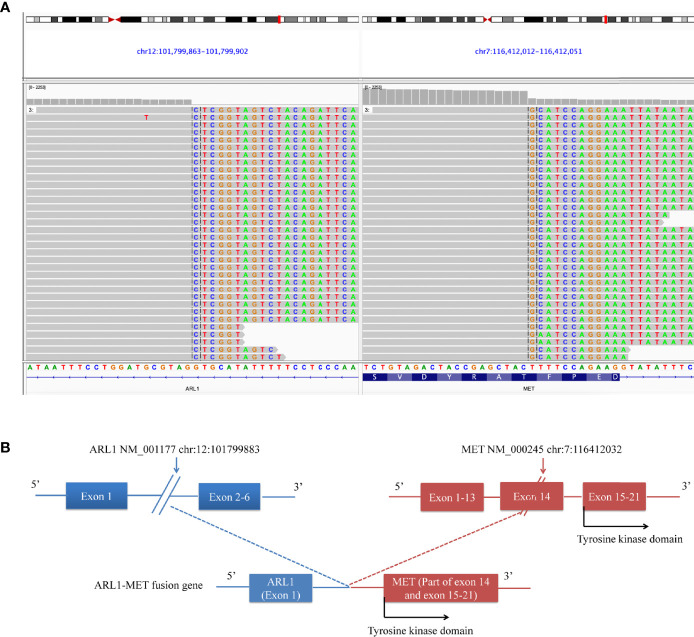
ARL1-MET fusion was detected in the patient. **(A)** Sequencing reads of the ARL1-MET fusion revealed by the Integrative Genomics Viewer (IGV). **(B)** Illustration of ARL1-MET fusion.

**Table 1:** Mutation profile of right lung tumor in the patient before treatment with crizotinib.

Gene	Transcript	Exon	Nucleotide change	Alteration	Mutant allele frequency
*ARL1-MET* fusion	ARL1:NM_001177	1	ARL1 (Exon1)-MET (Exon14)		74.27%
	MET : NM_000245	15			
*TP53*	NM_000546	8	c.811G>T	p.Glu271*	42.39%
*AKT1*	NM_001014432	6	c.428A>C	p.His143Pro	45.37%
*CCND1*	NM_053056	1	c.94C>G	p.Leu32Val	49.08%

* a nonsense mutation in a sequence of DNA that results in a premature stop codon.

To clarify the mutation profile of adenocarcinoma in the left lung, FFPE sections of tumor tissues resected from the left lung of the patient were subjected to NGS using a 500-gene panel (CSO 500, ChosenMed Technology (Beijing) Co. Ltd., Beijing, China) on September 28, 2021. The average sequencing depth was 347.4×. The results of NGS analysis are shown in **Table 2**. Using a combination of pathological analysis and mutation profile, SMPLC was diagnosed in the patient (**Figures 1A, B**; **Tables 1**, **2**).

**Table 2:** Mutation profile of left lung tumor in the patient.

Gene	Transcript	Exon	Nucleotide change	Alteration	Mutant allele frequency
*CCND1*	NM_053056	1	c.94C>G	p.L32V	43.08%
*FLI1*	NM_002017	2	c.196G>A	p.V66I	44.52%
*ERBB2*	NM_004448	20	c.2313_2324dup	p.772_775dup	20.66%
*MED12*	NM_005120	36	c.4897G>T	p.E1633*	3.82%

* a nonsense mutation in a sequence of DNA that results in a premature stop codon.

**Figure 3:**
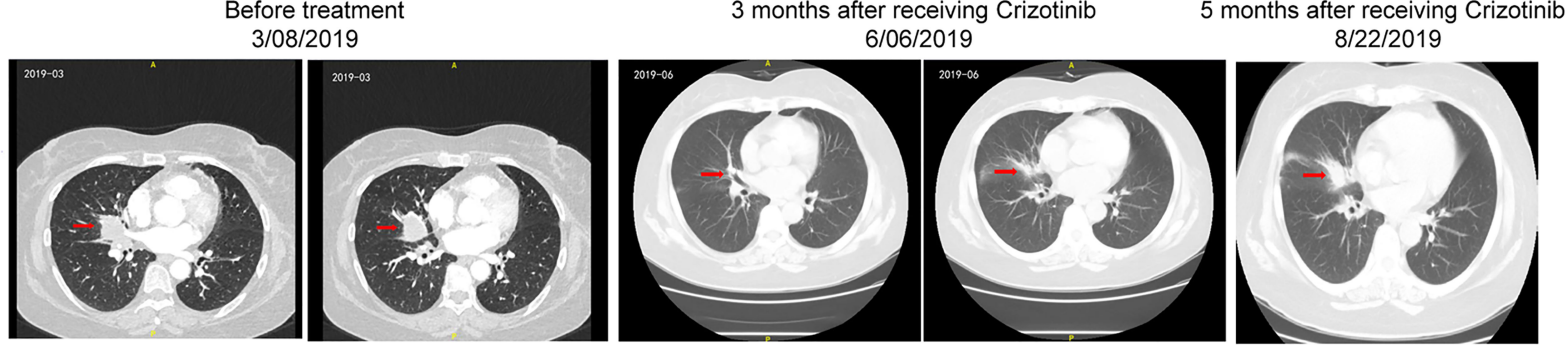
Dynamic imaging of the soft tissue density nodule in the right middle lobe and near the hilum during the treatment with crizotinib.

**Figure 4:**
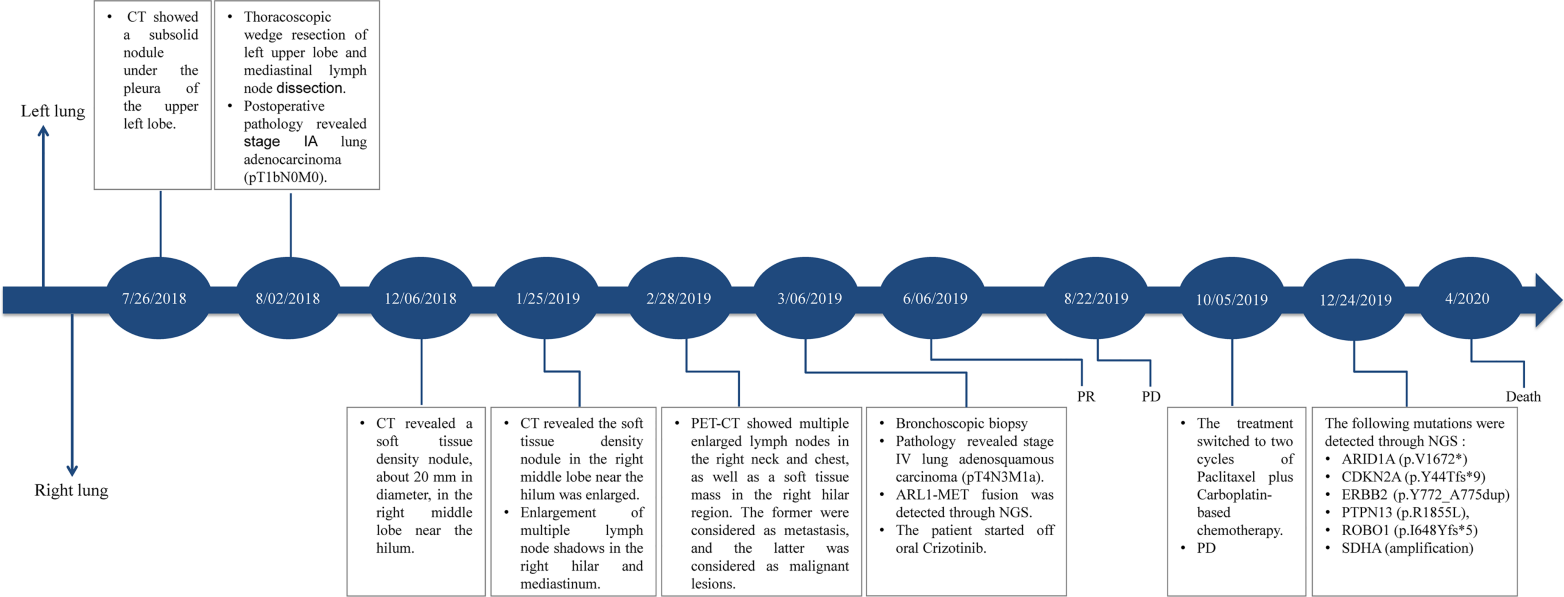
Case timeline. * a premature stop codon due to frameshift mutation or deletion mutation.

The patient was started on off-label crizotinib treatment (p.o., b.i.d.). Three weeks after treatment with crizotinib, cough and dyspnea were remarkably relieved. In June 2019, thoracic CT showed that the size of the nodule shadow in the right middle lobe was reduced to approximately 22 mm × 14 mm, and multiple lymph node shadows in the right pulmonary hilum and mediastinum were also greatly reduced. She achieved a partial response (PR) defined by RECIST1.1 (**Figure 3**). However, by August 22, 2019, the patient’s disease had progressed rapidly, and there was no improvement even after two cycles of paclitaxel plus carboplatin-based chemotherapy. A second biopsy was performed on December 24, 2019 to clarify the underlying mechanism of resistance to crizotinib. Pathology revealed that the adenosquamous carcinoma in the right lung had transformed into squamous carcinoma (**Figure 1B**). FFPE sections of tumor tissues were subjected to NGS through an 825-gene panel (Genetron Health (Beijing) Co. Ltd., Beijing, China) with an average sequencing depth of 1,609.93×. The results revealed that the original *ARL1-MET* fusion disappeared but *ERBB2* insertion (NM_004448: exon 20: c.2313_2324dup: p.Y772_A775dup) appeared (**Table 3**). The patient declined any inhibitors against ERBB2 insertion for personal reasons. She died 6 months later. The case timeline is shown in **Figure 4**.

**Table 3:** Mutation profile of right lung tumor in the patient after resistance to crizotinib.

Gene	Transcript	Exon	Nucleotide change	Alteration	Mutant allele frequency/copy number
*ARID1A*	NM_006015	19	c.5014del	p.V1672*	2.3%
*CDKN2A*	NM_000077	1	c.130del	p.Y44Tfs*9	1.3%
*ERBB2*	NM_004448	20	c.2313_2324dup	p.Y772_A775dup	31.1%
*PTPN13*	NM_080683	34	c.5564G>T	p.R1855L	12.7%
*ROBO1*	NM_002941	14	c.1942del	p.I648Yfs*5	23.4%
*SDHA*	Gene amplification				2.6

* a premature stop codon due to frameshift mutation or deletion mutation.

## Discussion

In this case, a previously undescribed fusion of *ARL1* exon 1 (NM_001177) to part of *MET* exon 14 (NM_000245) was detected in a patient with NSCLC and SMPLC using NGS.

*ARL1* is located at 12q23.2, encoding a protein belonging to the ADP-ribosylation factor-like (ARL) family of proteins. *ARL1* is the first identified member of the large ARL family, which serves as an important regulator of the Golgi complex structure and is involved in several cellular processes, such as the endosomal trans-Golgi network, secretory trafficking, and innate immunity (9). *ARL1* has six exons.

The novel *ARL1-MET* fusion protein was predicted to contain exon 1 of *ARL1*, consisting of only one full codon. Thus, the fusion would essentially be a truncated version of *MET*, which preserved the complete tyrosine kinase domain of *MET* (**Figure 2B**). Histologically, the patient’s SMPLC was diagnosed as NSCLC. She underwent surgery for early malignancy in the left lung, and no recurrence was observed. Meanwhile, she received crizotinib for unresectable malignancies in the right lung and obtained a 5-month PFS.

In recent years, the carcinogenic effect of driving genes has been increasingly understood due to the in-depth study of NSCLC. Therefore, the routine treatment of patients with NSCLC has drastically changed. *MET* fusion is a rare structural change compared with oncogenic mutations and represents an increasing pool of druggable targets in NSCLC. Furthermore, compared with mutations and amplification of *MET*, gene rearrangements are less documented, and the therapeutic relevance of more complex structural rearrangements remains largely unknown (10, 11). *Translocated promoter region (TPR)-MET* fusion is one of the few known *MET* fusions. It is a rearrangement that fuses the oligomerization domain of *TPR* with the completed tyrosine kinase domain of *MET* maintaining its oncogenicity (12).

Because the patient’s sequencing results were negative for other oncogenic drivers, the *ARL1-MET* fusion may be the only potential druggable target. Crizotinib was the most appropriate choice for the patient, as it was initially developed as a *MET* inhibitor, and it has been proven to effectively inhibit *ALK* and *MET* among several other kinases (8), showing good efficacy in preclinical and phase I studies (13). Plenker et al. reported two cases of genomic rearrangements, leading to gene fusion of *KIF5B*, *STARD3NL*, and *MET*. Both patients obtained a PR to crizotinib (1). Davies et al. reported a significant response to crizotinib in a patient with NSCLC positive for an *HLA-DRB1-MET* fusion (2). Our results provide evidence that novel *ARL1-MET* fusion may be an appealing target in patients with NSCLC.

MET tyrosine kinase inhibitors (MET-TKIs) are generally divided into three types depending on their structure and modes of binding with MET. Types I and II are ATP-competitive inhibitors. Type I inhibitors are further subdivided into types Ia and Ib, depending on their interaction with the solvent front residue G1163. Type Ia inhibitors, such as crizotinib, interact with the solvent front residue G1163, whereas type Ib inhibitors, such as capmatinib, tepotinib, savolitinib, and APL-101, are independent of G1163 interaction. Type II MET-TKIs, such as cabozantinib, foretinib, merestinib, and glesatinib, are ATP competitors that bind to the inactive “DFG out” conformation of MET. Type III MET-TKIs, such as tivantinib, are non-ATP-competitive allosteric inhibitors (14, 15). Among these MET-TKIs, crizotinib, capmatinib, and tepotinib are recommended by the Version 1, 2022 of the NCCN Guidelines for NSCLC for the patients with advanced NSCLC harboring *MET* exon 14 skipping mutations or high-level *MET* amplification. Also, capmatinib and tepotinib have been approved by the Food and Drug Administration (FDA) of the United States for the patients with metastatic NSCLC harboring *MET* exon 14 skipping mutations. Others are being evaluated in clinical trials.

Studies have identified two potential mechanisms underlying resistance to MET-TKIs. One was a secondary mutation of some active sites of *MET*, like a point mutation at the tyrosine residue Y1230 of the MET tyrosine kinase domain and somatic M1268T mutation (16, 17). The other was activation of the downstream signaling pathways, including the RAS-MAPK, PI3K-AKT, and STATs pathways. NGS through the second right lung biopsy revealed that the original *ARL1-MET* fusion had “disappeared”. Through pathology analysis, we speculate that the adenocarcinoma component harboring the *ARL1-MET* fusion had shrunk under treatment, leaving only the squamous component to grow. Thus, the novel *ARL1-MET* fusion did not disappear, but rather it was not present in the squamous tumor cells. This might explain the strikingly different mutation profiles in the tumors before and after treatment. However, *ERBB2* insertion (p.Y772_A775dup) appeared which activates the bypass signaling pathway. This might be a mechanism underlying resistance in the patient (18).

There are a few limitations to our study. First, whether this finding means that all of the products of *MET* fusions could serve as oncogenic drivers in NSCLC remains uncertain. Second, it is still unclear why the patient rapidly developed resistance to crizotinib after 5 months of remission. Finally, there was only one case reported in our study, and more cases positive for *ARL1-MET* fusion are needed in the future to confirm the efficacy of crizotinib.

## Conclusion

In conclusion, to the best of our knowledge, this study provides the first clinical evidence that the novel *ARL1-MET* fusion might be an actionable alteration in NSCLC. Further studies are needed to understand and overcome the potential mechanism(s) associated with resistance to MET inhibitors.

## Data Availability Statement

The original contributions presented in the study are included in the article/supplementary materials. Further inquiries can be directed to the corresponding author.

## Ethics Statement

The studies involving human participants were reviewed and approved by the ethics committee of Tianjin Medical University General Hospital. The patients/participants provided their written informed consent to participate in this study.

## Author Contributions

Conceptualization: DZ. Attending physicians for the patient: LK. Writing—original draft: QM. Editing draft: DZ. All authors read and approved the final manuscript.

## Conflict of Interest

The authors declare that the research was conducted in the absence of any commercial or financial relationships that could be construed as a potential conflict of interest.

## Publisher’s Note

All claims expressed in this article are solely those of the authors and do not necessarily represent those of their affiliated organizations, or those of the publisher, the editors and the reviewers. Any product that may be evaluated in this article, or claim that may be made by its manufacturer, is not guaranteed or endorsed by the publisher.
